# Reprogramming of Histone H3 Lysine Methylation During Plant Sexual Reproduction

**DOI:** 10.3389/fpls.2021.782450

**Published:** 2021-11-30

**Authors:** Huihui Fang, Yuke Shao, Gang Wu

**Affiliations:** State Key Laboratory of Subtropical Silviculture, Laboratory of Plant Molecular and Developmental Biology, Collaborative Innovation Center for Efficient and Green Production of Agriculture in Mountainous Areas of Zhejiang Province, College of Horticulture Science, Zhejiang Agriculture and Forestry University, Hangzhou, China

**Keywords:** H3 lysine methylation reprogramming, plant sexual reproduction, histone lysine methyltransferases, histone methylation readers, histone demethylases, *Arabidopsis*

## Abstract

Plants undergo extensive reprogramming of chromatin status during sexual reproduction, a process vital to cell specification and pluri- or totipotency establishment. As a crucial way to regulate chromatin organization and transcriptional activity, histone modification can be reprogrammed during sporogenesis, gametogenesis, and embryogenesis in flowering plants. In this review, we first introduce enzymes required for writing, recognizing, and removing methylation marks on lysine residues in histone H3 tails, and describe their differential expression patterns in reproductive tissues, then we summarize their functions in the reprogramming of H3 lysine methylation and the corresponding chromatin re-organization during sexual reproduction in *Arabidopsis*, and finally we discuss the molecular significance of histone reprogramming in maintaining the pluri- or totipotency of gametes and the zygote, and in establishing novel cell fates throughout the plant life cycle. Despite rapid achievements in understanding the molecular mechanism and function of the reprogramming of chromatin status in plant development, the research in this area still remains a challenge. Technological breakthroughs in cell-specific epigenomic profiling in the future will ultimately provide a solution for this challenge.

## Introduction

Histones are the basic packing and organizing proteins in eukaryotic nuclei that package genomic DNA into nucleosomes, the basic repeating structural unit of the higher-order chromatin. The nucleosome comprises 146 base pairs of DNA wrapped in 1.7 superhelical turns around a histone octamer, which contains two copies of each core histone protein, H2A, H2B, H3, and H4. Although the H1 protein itself does not form part of the nucleosome (Luger et al., 1997, 2012), it acts as a linker histone to stabilize inter-nucleosomal DNA. The amino-terminal tails of histone proteins are subject to various types of posttranslational modifications at specific residues, including methylation, acetylation, ubiquitination, phosphorylation, and sumoylation (De Lucia et al., 2008; Liu et al., 2010; Weinhofer et al., 2010; de la Paz et al., 2015; Borg et al., 2020; Ryu and Hochstrasser, 2021). Histone modifications affect nucleosome packaging, and then the chromatin status and gene transcriptional activity depending on the site and degree of specific modification (Bastow et al., 2004; Alvarez-Venegas and Avramova, 2005; Hiragami-Hamada et al., 2016). Dynamic regulation of histone modification has been shown to be tightly linked to a variety of developmental processes in both plants and animals (Greer and Shi, 2012; Zhao et al., 2019; Cheng et al., 2020).

Plant sexual reproduction involves two major processes: the meiosis and the following fertilization (Wang and Copenhaver, 2018). The reproductive lineage in plants is established late in floral development, which is opposite to the early germline determination during embryogenesis in most animals (Feng et al., 2010). During plant sexual reproduction, a variety of epigenetic memories and chromatin modifications acquired in response to both developmental and environmental cues before the establishment of reproductive lineage need to be reprogrammed to ensure the integrity of genetic information between generations (Borg et al., 2020; Ono and Kinoshita, 2021). Reprogramming of histone methylation during plant sexual reproduction has been shown to be required for resetting the chromatin status toward pluri- or totipotency in gametes and the zygote, thus ensuring to establish new cell fates during plant sexual reproduction (Feng et al., 2010; Gutierrez-Marcos and Dickinson, 2012; Kawashima and Berger, 2014; She and Baroux, 2014; Borg et al., 2020).

In this review, we start by introducing enzymes required for writing, reading, and removing H3 lysine methylation marks, and then we describe their expression patterns in reproductive tissues, including flower bud, inflorescence, anther, stamen, pollen, ovule, embryo, endosperm, and siliques, and finally, we discuss the reprogramming of H3 lysine methylation during plant sexual reproduction and their biological significance on the establishment of new cell fate, with a particular emphasis on the epigenomic resetting of chromatin status toward gamete pluripotency and zygote totipotency.

## “Writers,” “Readers,” and “Erasers” for Histone H3 Lysine Methylation

**FIGURE 1:**
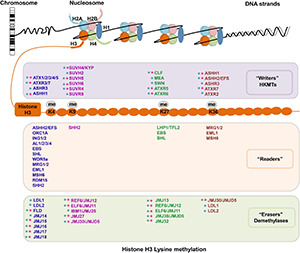
Writers, readers, and erasers of H3 lysine methylation in *Arabidopsis*. The identified histone methyltransferases “writers,” binding effectors “readers” and demethylases “erasers” for H3K4, H3K9, H3K27, and H3K36 methylation (me) are summarized in different colored boxes, respectively. The writers are summarized in the light purple box, the readers are summarized in the light orange box, and the erasers are summarized in the light green box. Moreover, H3 methylation associated enzymes acting on different residues are listed in different colors. The writers, readers and erasers for H3K4 methylation are listed in blue words, the enzymes for H3K9 methylation are listed in purple words, the enzymes for H3K27 methylation are listed in green words, and enzymes for H3K36 methylation are listed in brown words. The catalytic specificity for mono-, di-, and trimethylations is not distinguished. Moreover, we added different colored asterisks to discriminate enzymatic activity *in vitro* and deduced from loss-of-function mutants. The pink asterisk represents this enzyme has corresponding enzymatic activity *in vitro*, while the cyan asterisk represents the function of this enzyme was demonstrated by loss-of-function mutant.

Histone H3 methylation can occur at various sites, but primarily on lysine (Lys, K) residues, and the K4, K9, K27, and K36 residues on H3 tails can be mono-, di-, and/or trimethylated (Liu et al., 2010). H3 lysine methylation is an important and complex epigenetic mark that decorates both transcriptionally silenced and active chromatin status, depending on the specific sites and degrees of methylation (Greer and Shi, 2012; Atlasi and Stunnenberg, 2017; Samo et al., 2021). Typically, H3K4 and H3K36 methylations are linked to the transcriptionally active chromatin status, while H3K9 and H3K27 methylations correlate with heterochromatinization and transcriptional inactivation (Liu et al., 2010; Cheng et al., 2020). The outcomes of H3 lysine methylation are dynamically regulated by “writers,” “readers,” and “erasers” of histone methylation. The SET (*S*uppressor of variegation, *E*nhancer of *Z*este and *T*rithorax) Domain Group (SDG) proteins are the main histone lysine methyltransferases (HKMTs), serving as “writers” for adding methylation marks to specific lysine residues on H3. Two types of histone demethylases (HDMs), the lysine-specific demethylase 1 (LSD1, or KDM1) homologs and Jumonji C (JmjC) domain-containing proteins (JMJs), act as “erasers” to remove methylation marks (Xiao et al., 2016; Cheng et al., 2020). Additionally, distinct H3 lysine methylation modifications can recruit specific binding effectors, namely “readers,” to recognize histone marks and mediate downstream biological events, including chromatin organization and gene transcriptional regulation (Berger, 2007; Zhao et al., 2018). Here, we introduce some most commonly known “writers,” “readers,” and “erasers” for H3 lysine methylation (Figure 1) based on their targeted residues.

### “Writers” for Adding Methylation Marks on H3 Lysine Residues

The SDG proteins serve as “writers” for adding H3 lysine methylation marks, and the SET domain is responsible for the catalytic activities of HKMTs. At least 49 putative SET domain-containing proteins have been identified^1^ in *Arabidopsis* (Pontvianne et al., 2010; Cheng et al., 2020), and these SDG proteins can be divided into seven classes (Class I to Class VII) based on their domain architecture and/or difference in enzymatic activity, including Class I, the *E*(Z) (*E*nhancer of *Z*este) homologs; Class II, the ASH1 (Absent, Small, or Homeotic discs 1) groups [ASH1 homologs (ASHH) and ASH1-related proteins (ASHR)]; Class III, the Trx (*T*rithorax) groups (TRX homologs and TRX-related proteins); Class IV, ATXR5 (Arabidopsis *T*rithorax-related 5) and ATXR6, which only existed in yeast and plants, are separated from the TRX subfamily and considered to be a newly separated IV subfamily (Zhou et al., 2020); Class V, the SU(VAR)3-9 sub-groups [SU(VAR)3-9 homologs (SUVH) and SU(VAR)3-9 related proteins (SUVR)] (Pontvianne et al., 2010; Cheng et al., 2020). Additionally, a series of proteins containing the split SET domains were incorporated into the SDG proteins and were further classified into VI (SMYD) and VII (SETD) subfamilies (Springer et al., 2003; Ng et al., 2007). The genetic information and classification of SDG proteins are summarized in Table 1 based on the specific residues on which they act, and their functions in mediating mono-, di-, or tri-methylation modification on H3K4, H3K9, H3K27, and H3K36 sites are discussed subsequently.

**TABLE 1:** Summary of key writers, readers, and erasers for H3 lysine methylation.

	**Writers**				**Readers**			**Erasers**		
	
**Residue sites**	**Formal name**	**ChromDB ID**	**Gene locus**	**Class**	**Protein**	**Gene locus**	**Binding domain**	**Enzymes**	**Gene locus**	**Class**
**H3K4**	ATX1	SDG27	At2g31650	Class III TrxG-SDG	ORC1A	At4g14700	PHD domain	FLD	At3g10390	LSD1
	ATX2	SDG30	At1g05830		ING1/2	At3g24010/At1g54390		LDL1	At1g62830	
	ATX3	SDG14	At3g61740		AL1/2/3/4	At5g05610/At3g11200/At3g42790/At5g26210		LDL2	At3g13682	
	ATX4	SDG16	At4g27910		EBS	At4g22140	PHD-BAH	JMJ14	At4g20400	KDM5/JARID1-JMJs
	ATX5	SDG29	At5g53430		SHL	At4g39100		JMJ15	At2g34880	
	ATXR3	SDG2	At4g15180		WDR5a	At3g49660	WD40 repeats	JMJ16	At1g08620	
	ATXR7	SDG25	At5g42400		MRG1/2	At4g37280/At1g02740	Chromo domain	JMJ17	At1g63490	
	ASHR3	SDG4	At4g30860	Class II ASH1-SDG	EML1	At3g12140	Tudor domain	JMJ18	At1g30810	
	ASHH1	SDG26	At1g76710		MSH6	At4g02070				
					RDM15	At4g31880				
					ASHH2/EFS/SDG8	At1g77300	CW domain			
					SHH2	At3g18380	Zinc Finger CW domain			
**H3K9**	SUVH4/KYP	SDG33	At5g13960	Class V Su(var)-SDG	SHH2	At3g18380	Zinc Finger CW domain	IBM/JMJ25	At3g07610	KDM3/JHDM2-JMJs
	SUVH2	SDG3	At2g33290					JMJ27	At4g00990	
	SUVH5	SDG9	At2g35160					JMJ30/JMJD5	At3g20810	JmjC-domain only-JMJs
	SUVH6	SDG23	At2g22740					ELF6/JMJ11	At5g04240	KDM5/JARID1-JMJs
	SUVR4	SDG31	At3g04380					REF6/JMJ12	At3g48430	
	SUVR5	SDG6	At2g23740							
**H3K27**	ATXR5	SDG15	At5g09790	Class IV	LHP1/TFL2	At5g17690	Chromo domain	ELF6/JMJ11	At5g04240	KDM5/JARID1-JMJs
	ATXR6	SDG34	At5g24330		EBS	At4g22140	PHD-BAH	REF6/JMJ12	At3g48430	
	CLF	SDG1	At2g23380	Class I E(Z)-SDG	SHL	At4g39100		JMJ13	At5g46910	
	SWN	SDG10	At4g02020					JMJ30/JMJD5	At3g20810	JmjC-domain only-JMJs
	MEA	SDG5	At1g02580					JMJ32	At3g45880	
**H3K36**	ASHH2/EFS	SDG8	At1g77300	Class II ASH1-SDG	MRG1/2	At4g37280/At1g02740	Chromo domain	JMJ30/JMJD5	At3g20810	JmjC-domain only-JMJs
	ASHH1	SDG26	At1g76710		MSH6	At4g02070	Tudor domain	LDL1	At1g62830	LSD1
	ASHR3	SDG4	At4g30860		EML1	At3g12140		LDL2	At3g13682	
	ATXR7	SDG25	At5g42400	Class III TrxG-SDG						
	ATXR2	SDG36	At3g21820	Class VI SMYD						

H3K4 methylation is mainly enriched in genic regions but depleted in transposons. H3K4me1 accumulates mainly in the transcribed regions, while H3K4me2/3 are enriched in the promoter and 5′ end of the transcribed regions, with H3K4me3 peaking slightly upstream of H3K4me2 (Zhang et al., 2009; Cheng et al., 2020). H3K4me1 and H3K4me2 associate with both active and inactive transcription, whereas H3K4me3 strongly links to transcriptional activation (Cheng et al., 2020). H3K4me1 is highly correlated with CG DNA methylation in the transcribed regions of genes, but H3K4me2/3 and DNA methylation appear to be mutually exclusive. TrxG-SDG proteins function as the main writers for adding H3K4 methylation marks. The TrxG-SDG members ATX1/SDG27 and ATX2/SDG30 are two chromosomal duplications in *Arabidopsis* with divergent functions in catalyzing H3K4 methylation. ATX1/SDG27 has the H3K4 methyltransferase activity and mainly catalyzes H3K4me3 deposition (Alvarez-Venegas et al., 2003), while ATX2/SDG30 is responsible for H3K4me2 (Saleh et al., 2008). This is further confirmed in the *axt1* mutant with reduced H3K4me3 deposition and unchanged H3K4me2 level at the *FLOWERING LOCUS C* (*FLC*) locus. Additionally, ATX1 plays two distinct roles in regulating target genes transcription by facilitating TATA binding protein (TBP) and RNA Polymerase II (Pol II) occupancy at promoters and depositing H3K4me3 within the transcribed region (Ding et al., 2011). ATX3/SDG14, ATX4/SDG16, and ATX5/SDG29 redundantly contribute to both H3K4me2 and H3K4me3 (Chen et al., 2017). ATXR3/SDG2 is the major methyltransferase to catalyze H3K4me3 and plays crucial roles in both sporophyte and gametophyte development (Berr et al., 2010; Guo et al., 2010; Yun et al., 2012; Foroozani et al., 2021). In addition, ATXR3/SDG2 can be co-recruited with a Swd2-like COMPASS axillary subunit onto most transcribed genes to increase H3K4me3 occupancy, which is an atypical and H2B ubiquitination independent pathway (Fiorucci et al., 2019). ATXR7/SDG25 is responsible for all three types of H3K4 methylation (Tamada et al., 2009). In addition, two ASH1-SDG proteins, ASHR3/SDG4 and ASHH1/SDG26 also contribute to H3K4 methylation. ASHR3/SDG4 mediates H3K4me2/3 and H3K36me3 deposition to regulate pollen tube growth and stamen development (Cartagena et al., 2008); ASHH1/SDG26 (Berr et al., 2015) plays roles in both H3K4 and H3K36 methylation. Loss-of-function of ASHH2/EFS/SDG8 reduced H3K4me3 and increased H3K4me2 levels surrounding the CAROTENOID ISOMERASE (CRTISO) translation start site, indicating that ASHH2/EFS/SDG8 regulates H3K4 methylation at least at single or certain genes (Cazzonelli et al., 2009). However, the global profiling of histone methylation demonstrated that the H3K4me3

profiles were comparable between *sdg8* and WT, while H3K36me3 was significantly reduced in *sdg8*, indicating that ASHH2/EFS/SDG8 is mainly responsible for H3K36me3 (McLaughlin et al., 2014; Li et al., 2015).

H3K9me1 and H3K9me2 histone marks are repressive modification marks enriched at chromocenters (Jackson et al., 2004). H3K9me2 is mainly enriched in transposons and repeated sequences, consistent with its primary role in repressing transposon activities and silencing some repeated sequences. Surprisingly, H3K9me3 is mainly associated with euchromatin and transcribed genes, although low levels of this mark are also detected at transposons and repetitive sequences (Mathieu et al., 2005; Charron et al., 2009; Cheng et al., 2020). H3K9 methylation is catalyzed by members of the Su(var)3-9 group proteins. SUVH4/KYP/SDG33 is the first H3K9 methyltransferase to be identified, and it predominantly catalyzes H3K9me2 modification. In *kyp/suvh4* mutant, the deposition of H3K9me2 in heterochromatin is greatly reduced, but H3K9me1 is not affected significantly, implying that SUVH4/KYP/SDG33 has little contribution to H3K9me1 (Liu et al., 2010). SUVH4/KYP/SDG33 is also required for the maintenance of DNA methylation by binding to the methylated cytosines, providing a link between histone modification and DNA methylation (Jackson et al., 2002; Du et al., 2014). SUVH2/SDG3 has no H3K9 methylase activity *in vitro* (Liu et al., 2014), but H3K9me1 level is significantly reduced in the *suvh2* null mutants (Naumann et al., 2005), suggesting an indirect role of SUVH2/SDG3 in regulating H3K9 methylation. SUVH5/SDG9 and SUVH6/SDG23, two homologs of SUVH4/KYP/SDG33, catalyze H3K9me1 and H3K9me2 mainly in transposons and repetitive sequences (Ebbs et al., 2005; Ebbs and Bender, 2006). SUVR4/SDG31 (Thorstensen et al., 2006) and SUVR5/SDG6 (Caro et al., 2012) preferentially mediate the deposition of H3K9me1 and H3K9me2, respectively. The role of other Su(var)3-9 proteins in H3K9 methylation remains elusive.

H3K27 methylation is an important repressive histone modification mark in plants (Johnson et al., 2004; Feng and Jacobsen, 2011). H3K27me1 accumulates significantly in constitutively silenced heterochromatin, which is of great significance in maintaining chromatin structure and transcriptional silencing (Jacob et al., 2009). Moreover, H3K27me1 deposition is independent of DNA methylation, which suggests that H3K27 methylation might have a distinct regulatory mechanism from H3K9 methylation. ATXR5/SDG15 and ATXR6/SDG34, the only two members of Class IV SDG proteins, contribute redundantly to the deposition of H3K27me1 (Jacob et al., 2009). H3K27me2 associates with both euchromatin and heterochromatin (Mathieu et al., 2005; Fuchs et al., 2006), while H3K27me3 is mostly restricted to the transcribed regions of genes to silence a large number of genes in *Arabidopsis* (Zhang et al., 2007a).

H3K27me3 is largely independent of DNA methylation and other epigenetic pathways, but is mainly dependent on Polycomb Repressive Complex 2 (PRC2) (Zhang et al., 2007a; Zheng and Chen, 2011; Xiao and Wagner, 2015). In *Arabidopsis*, members in the PRC2 complex contain three *E*(Z) homologs (CLF/SDG1, MEA/SDG5 and SWN/SDG10), three Su(z)12 homologs (FIS2, EMF2, and VRN2), five p55 homologs (MSI1-5), and only one Esc homolog (FIE) (Kohler et al., 2003). Alternative combinations of these members can form diverse PRC2 complexes, including FIS-PRC2, EMF2-PRC2, and VRN2-PRC2, that play various roles in different developmental processes (Kohler and Villar, 2008; Liu et al., 2010; Golbabapour et al., 2013). PRC1, which acts as a chromatin repressor through mediating the H2A mono-ubiquitination, can communicate with PRC2 (Kahn et al., 2016). VIVIPAROUS (VP1)/ABSCISIC ACID INSENSITIVE 3 (ABI3) LIKE protein (VAL) and Arabidopsis B lymphoma Moloney murine leukemia virus insertion region1 homolog (AtBMI1) mediated H2A ubiquitination initiates the repression of seed maturation genes, and this repression could be further maintained by PRC2-mediated H3K27me3 after the initiation (Yang et al., 2013), suggesting that the PRC1 activity is required for H3K27me3 deposition by PRC2 in certain cases. Another interesting case is that ALFIN1-like (AL) proteins can bind to H3K4me3 marks, and physically interact with PRC1 via its plant homeodomain finger (PHD) domain to form an AL-PHD-PRC1 complex to recruit the PRC2 complex to promote H3K27me3 deposition. This result has important implications for understanding the association between PRC1 and PRC2 complex, as well as the connection between H3K4me3 in gene activation and H3K27me3 in gene repression (Molitor et al., 2014). The bivalent bromo-adjacent homology (BAH)-PHD containing readers capable of recognizing two antagonistic histone marks, including EARLY BOLTING IN SHORT DAY (EBS) and the plant-specific histone reader SHORT LIFE (SHL), regulate floral transition by modulating their binding preference toward either H3K27me3 or H3K4me3 to provide a distinct mechanism of interaction between active and repressive chromatin status (Qian et al., 2018; Yang et al., 2018). Transcription factors (TFs), such as Class I BASIC PENTACYSTEINE (Class I BPC) and C1-2iD ZnF TFs, recruit and interact with PRC2 physically by binding to the Polycomb Response Elements (PREs) (Xiao et al., 2017; Zhou Y. et al., 2018). The telomere-repeat-binding factors (TRBs) recruit CLF/SWN-PRC2 through the telobox-related motifs in *Arabidopsis* (Zhou Y. et al., 2018). A transcriptional repressor, TEMPRANILLO 1 (TEM1), recognizes the 5′-UTR sequence of the *FLOWERING LOCUS T* (*FT*) gene to recruit PRC2 to regulate floral transition (Hu et al., 2021). Recent studies show that two transcriptional repressors, VAL1 and VAL2, are required for PRC2 recruitment for target silencing in *Arabidopsis* (Fouracre et al., 2021; Yuan et al., 2021). TFs with an ethylene-responsive element binding factor-associated amphiphilic repression (EAR) domain can trigger both histone deacetylase complex and PRC2 activities, and different TFs have an additive effect on PRC2 activity (Baile et al., 2021). In the future, it will be of great interest to identify novel proteins required for PRC2 recruitment to a specific locus to regulate gene repression.

H3K36me2 and H3K36me3 are two predominant patterns of H3K36 methylation because H3K36me1 exists simply as a precursor of H3K36me2/3 in plants (Cheng et al., 2020). H3K36 methylation associates with transcriptional activation and transcriptional elongation. H3K36 methylation is specifically mediated by the ASH1-SDG proteins. ASHH2/EFS/SDG8 is the major H3K36 methyltransferase *in vivo*, and is mainly responsible for H3K36me2/3 (Dong et al., 2008; Xu et al., 2008). It has been reported that ASHH2/EFS/SDG8 can physically interact with the C-terminal domain of the RNA Pol II to facilitate transcription elongation, providing a possible link between Pol II loading and H3K36 methylation deposition (Zhong et al., 2019; Zhang X. et al., 2020). ASHH1/SDG26 has the *in vitro* methyltransferase activity on oligo-nucleosomes, and might repress some gene transcription in an indirect manner (Xu et al., 2008). ASHR3/SDG4 catalyzes H3K36me3 and H3K4me2 to regulate pollen tube growth as their levels were dramatically reduced in the vegetative nuclei in *sdg4* mutant pollen (Cartagena et al., 2008). In addition, although the TrxG-SDG member ATXR7/SDG25 is primarily responsible for H3K4 methylation, it can also catalyze H3K36me2 to activate *FLC* expression to repress flowering (Berr et al., 2009). Moreover, ATXR2/SDG36, a member of the Class VI SMYD subfamily, promotes the accumulation of H3K36me3 during callus formation (Lee et al., 2017).

### “Readers” for Recognizing H3 Lysine Methylation Marks

Histone marks can be recognized by specific domains of the effector proteins, referred to as “readers.” There are several types of domains that can recognize and bind to H3 lysine methylation marks: the PHD domain, the WD40 repeats (Jiang et al., 2009), and the “Royal Family” domains, which include the Chromo domain, the Tudor domain, the conserved Pro-Trp-Trp-Pro motif (PWWP) malignant brain tumor (MBT) domain, and the plant Agenet module (Zhao et al., 2018). Some readers either contain a single domain, or multiple domains to interact with other factors in macromolecular complexes (Cheng et al., 2020; Xu and Jiang, 2020).

In *Arabidopsis*, a number of proteins have been confirmed to bind to the methylated H3 to generate specific downstream nuclear processes. ORC1, the large subunit of origin-recognition complex (ORC), interacts with the H3K4me3 mark by its PHD finger domain to activate the transcription of target genes (de la Paz and Gutierrez, 2009). The PHD finger containing proteins ING1, ING2, and AL family members, were also shown to be the H3K4me2/3 readers. AL1, AL2, and AL4 have higher affinities to bind to H3K4me3 than H3K4me2, while AL3 has similar binding affinities toward H3K4me2 and H3K4me3 (Zhao et al., 2018). The EBS contains bivalent BAH-PHD reader modules that bind to either H3K27me3 or H3K4me3, and acts as a reader to switch binding between H3K27me3 and H3K4me3, thus timely regulating *FLC* transcription and floral transition (Yang et al., 2018). A plant-specific histone reader SHL can also recognize both H3K27me3 and H3K4me3 via its BAH and PHD domain, and BAH-H3K27me3 and PHD-H3K4me3 interactions are important for SHL-mediated floral repression (Qian et al., 2018).

*Arabidopsis* WD40-repeat 5a (WDR5a) binds to the K4-methylated H3 tail of FRIGIDA (FRI) specifically to enrich the WDR5a-containing COMPASS-like complex and H3K4 methylation at the *FLC* locus (Jiang et al., 2009). The single chromo domain of LHP1/TFL2 recognizes specifically H3K27me3 marks and represents a potential stabilizing factor of PRC2 activity (Turck et al., 2007; Zhang et al., 2007b). Morf Related Gene (MRG) group proteins, MRG1 and MRG2, bind to H3K4me3 and H3K36me3 peptides through their chromo domains to regulate *FT* transcription and flowering time (Xu et al., 2014; Zy et al., 2014). In *Arabidopsis*, the single Tudor domain protein EMSY-like 1 (EML1) functions as a plant-specific H3K4me2/3 reader, different from the case in humans that only double or tandem Tudor domains can recognize H3K4me2/3 (Zhao et al., 2018), indicating a plant-specific recognition mode. EML1 can also recognize H3K36me3 (Milutinovic et al., 2019) with a much weaker binding affinity for H3K36me3 than for H3K4me3 (Zhao et al., 2018). The Tudor domain protein MSH6, a DNA mismatch repair protein, binds to H3K4me3 with a much weaker affinity than H3K36me3 *in vitro* (Zhao et al., 2018). Recently, it has been reported that RDM15 with a Tudor domain specifically recognizes the H3K4me1 mark, and it functions as an RNA-directed DNA methylation (RdDM) component, thus establishing a link between H3K4me1 and RDM15-mediated RdDM (Niu et al., 2021). The Zinc Finger CW domain of SAWADEE homeodomain homolog 2 (SHH2) has a strong binding affinity for H3K4me3 (Zhao et al., 2018), and the maize SHH2 can specifically recognize H3K9me1 via its SAWADEE domain to establish a functional link between the RdDM pathway and H3K9me1 modification (Wang et al., 2021). The CW domain of ASHH2/EFS/SDG8 exhibits binding preference to H3K4me1, which is different from the mammalian counterpart that has binding preference to H3K4me3 (Liu and Huang, 2018).

Collectively, recognition of a distinct histone mark by a corresponding reader, the histone “mark-reader” pair, indicates a general “*trans*-acting” epigenetic regulatory mechanism in plants (Roudier et al., 2009; Zhao et al., 2018). However, the phenomenon that one reader can simultaneously recognize two or even more histone marks might provide an important mechanism for plants to achieve different biological readouts by modulating the binding affinities of one reader toward multiple marks. Identification of more histone “mark-reader” pairs will be a future challenge.

### “Erasers” for Removing Methylation Marks on H3 Lysine Residues

Two types of demethylases, the lysine-specific demethylase 1 (LSD1, or KDM1) homologs and Jumonji C (JmjC) domain-containing proteins (JMJs), contribute to the removal of H3 lysine methylation marks at different sites by interacting with different cofactors (Liu et al., 2010; Xiao et al., 2016). The KDM1/LSD1 demethylases have demethylase activities on di- and mono-methylated lysines, but not on tri-methylated lysines (Klose and Zhang, 2007). The KDM1/LSD1 homologs LDL1, LDL2, and FLD function to remove H3K4me2 marks. Both H3K4me2 and H3K4me3 deposition are elevated at the *FLC* and *FWA* loci in *ldl1ldl2* and *fld* mutants (Jiang et al., 2007; Liu et al., 2007; Shafiq et al., 2014; Berr et al., 2015). Moreover, the increased levels of both H3K4me2/3 and H3K36me3 at *FLC*, *FT*, *MAF2*, *MAF4* and *MAF5* in *ldl1ldl2* mutant suggest the functions of LDL1 and LDL2 in removing H3K36me3 in addition to H3K4me2/3. A large number of JMJs proteins can act on mono-, di-, and tri-methylated lysines, and they can be divided into five subgroups based on their sequence similarity: the KDM5/JARID1 group, the KDM4/JHDM3 (JmjC domain-containing histone demethylase 3) group, the KDM3/JHDM2 group, the JMJD6 group, and the JmjC domain-only group (Lu et al., 2008; Liu et al., 2010). Among the KDM5/JARID1 proteins, JMJ14/15/16 act on all three types of methylated H3K4 (Lu et al., 2010; Yang et al., 2012; Liu P. et al., 2019; Liu Y. et al., 2019), while JMJ18 can only demethylate H3K4me2/3 (Yang et al., 2012). JMJ27, a member of the KDM3/JHDM2 proteins, demethylates H3K9me1/2 to regulate flowering (Dutta et al., 2017). As a homolog of human KDM3/JHDM2, IBM1/JMJ25 demethylates H3K9me1/2 and prevents the spread of H3K9me2 at loci near TEs and repetitive elements (Saze et al., 2008; Miura et al., 2009). JMJ13, a member in the KDM4/JHDM3 subfamily, acts as an eraser to remove H3K27me3 deposition at the *FT* locus (Zheng et al., 2019). ELF6 and REF6, the other members of this subfamily, erase H3K27me2/3 methylation redundantly during plant development (Yu et al., 2008; Li et al., 2016). In addition, H3K9 methylation status at some loci can also be modulated by *elf6* and *ref6* loss-of-function mutations (Yu et al., 2008), indicating a potential link between the erasure of H3K9 and H3K27 methylation. Two JmjC domain-only proteins, JMJ30 and JMJ32, demethylate H3K27me2/3 jointly at the *FLC* locus to regulate flowering at elevated temperatures (Gan et al., 2014; Crevillen, 2020). JMJ30 can also act as an eraser to remove H3K36me2/3 (Yan et al., 2014) and H3K9me3 marks (Lee et al., 2018), suggesting multiple roles of a single demethylase in various lysine demethylation.

### Expression Patterns of “Writers,” “Readers,” and “Erasers” in Reproductive Tissues

**FIGURE 2:**
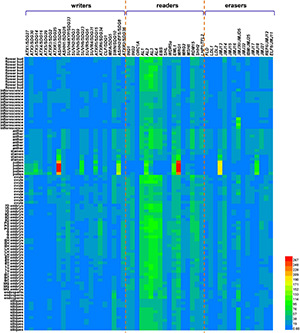
Heat map of differential expression patterns of H3 lysine methylation associated genes across various reproductive tissues in *Arabidopsis.* A total of 95 RNA-seq datasets from various tissues at different stages of plant sexual reproduction, including flower buds, inflorescence, anther, stamen, pollen, ovule, embryos at different developmental stages, endosperm, and siliques were collected and thoroughly analyzed and summarize here. The heat map shows the mRNA expression profile of genes, associated with writing, reading, and erasing the H3 lysine methylation marks during plant sexual reproduction. The symbols of genes are shown at the top of the heat map, and the tissue information are listed at the left side of the heatmap. The bar at the bottom right of the heat map represents relative expression values, which was presented based on the RNA fragments per kilobase of exon model per million mapped fragments, also known as the FPKM. 7D embryo, 7-day-old embryo; 8D embryo, 8-day-old embryo; PG embryo, Preglobular embryo; G embryo, Globular embryo; EH embryo, Early heart embryo; LH embryo, Late heart embryo; ET embryo, Early torpedo embryo; LT embryo, Late torpedo embryo; BC embryo, Bent cotyledon embryo; MG embryo, Mature green embryo. The red and blue denote highly and weakly expressed genes, respectively.

To investigate the effects of these “writers,” “readers,” and “erasers” on the reprogramming of H3 lysine methylation during sexual reproduction, we performed a thorough analysis of 95 RNA-seq datasets from various reproductive tissues, including flower buds, inflorescence, anther, stamen, pollen, ovule, embryos, endosperm, and siliques (Wolff et al., 2011; Loraine et al., 2013; Willmann et al., 2014; Klepikova et al., 2016; Tedeschi et al., 2017; Pignatta et al., 2018; Rahmati et al., 2018; Zhou M. et al., 2018; Hofmann et al., 2019). We summarized the expression patterns of these genes in reproductive tissues by using available information here, which might contribute to the understanding of their functions during plant reproduction. The datasets were downloaded from the Arabidopsis RNA-Seq Database (ARS) (Zhang H. et al., 2020), and the information of these RNA-seq libraries was summarized in Supplementary Table 1. Differential expression patterns of genes encoding “writers,” “readers,” and “erasers” in different sexual reproductive tissues were shown in the heat map (Figure 2), and the relative expression values were presented based on the RNA fragments per kilobase of exon model per million mapped fragments, also known as the FPKM. Some of these genes, including *ATX5*/*SDG29*, *ORC1A*, *JMJ15*, *JMJ32*, and *LDL2* have extremely low or even undetectable expression levels in all these reproductive tissues included, while some readers, including AL family members, EBS, EML1, MRG1, and RDM15, show higher transcript levels in almost all these reproductive tissues. In addition, some genes, including *ASHR3/SDG4*, *ATX3/SDG14*, *ASHH2/EFS/SDG8*, *SUVH6/SDG23*, *ATXR5/SDG15*, *MRG1*, *JMJ13*, *JMJ18*, and *EML1*, have much higher expression levels in pollen than in other tissues. Among them, *ASHR3/SDG4* is the first SDG gene identified to be associated with male sterility in *Arabidopsis*, and it is specifically expressed in open flowers, especially in the pollen, to induce pollen tube elongation. The *ashr3/sdg4* mutant has a larger number of infertile ovules (Cartagena et al., 2008; Zhou et al., 2020). ASHH2/EFS/SDG8 is required for normal anther differentiation, tapetum development and pollen maturation as approximately 90% of the pollen grains were aborted in *ashh2/efs/sdg8* mutant, and the expression of more than 600 genes associated with meiosis, tapetum development, and anther dehiscence was mis-regulated in *ashh2/efs/sdg8* inflorescences (Grini et al., 2009; Zhou et al., 2020). Additionally, ATXR3/SDG2-mediated H3K4me3 plays critical roles in gamete mitotic cell cycle progression and pollen vegetative cell function during male gametogenesis, and it acts indispensably for gametophyte chromatin landscape (Berr et al., 2010; Pinon et al., 2017). Our analysis might imply that the specific expression patterns of these genes in pollen may be of significance for pollen development and function by re-organization of histone modification. It was observed in a previous study that *FLD*-related GUS staining was observed in the anther-filament junction and in the tapetum, but not in the mature pollen grains by using *FLD* promoter-driven GUS transcriptional reporter transgenic lines (Martignago et al., 2019), which is consistent with our analysis that *FLD* is expressed in anther but not in pollen (Figure 2). The expression of *SUVH4/KYP/SDG33*, and *ATXR6/SDG34* exhibit extremely low levels in all these reproductive tissues except in developing embryos. Moreover, their expression declines gradually with embryo development, and few transcripts can be detected by RNA-seq in the mature embryos, indicating that some H3 lysine methylation-associated proteins may have more specific and crucial functions at a certain developmental stage. *SWN*, *AL1*, *AL2*, and *AL3* have higher expression levels in endosperm, which is a key evolutionary innovation of flowering plants, and has been identified as the site of genomic imprinting (Gehring and Satyaki, 2017). Correspondingly, the higher expression of SWN might function importantly in maintaining genomic imprinting. MEA/SDG5 has been well characterized, and it is a self-controlled imprinting gene to produce a cascade of parent-specific gene expression (Macdonald, 2012). Although transcriptomic data from central cells were not included in our RNA-seq dataset, studies have shown that MEA is specifically expressed in the late stage of the central cell (Luo et al., 2000), which is the second female gamete to initiate the endosperm lineage after fertilization. MEA loss-of-function leads to a large number of central cells proliferated excessively under unfertilized conditions, resulting in *mea* seeds with only endosperm but without embryos (Schmidt et al., 2013). Endosperm transfer cells (ETC) are one of the four main types of cells in the endosperm, and *AL1*, another gene highly expressed in the endosperm, has been shown to be an ETC-specific histone reader in rice (Kuwano et al., 2011; Lopato et al., 2014). Collectively, the tissue specific expression patterns of these genes imply their functional importance in these specific tissues. Moreover, it also indicates, from another perspective, that different H3 modification states and reprogramming in different tissues during sexual reproduction might have specific regulatory patterns by distinct enzymes. How these differential expression patterns of certain genes in different reproductive tissues link to their function in reprogramming of H3 lysine methylation during plant sexual reproduction remains an interesting research task.

## Reprogramming of H3 Lysine Methylation During Plant Sexual Reproduction

Haploid gametophyte generation in floral organs and the subsequent fertilization have great significance in the alternation of higher plant life cycle and the transgenerational transmission of genetic information (Dahia et al., 2020). Gametophyte generation, including sporogenesis and gametogenesis, involves a series of cell division and differentiation, and the consequent zygote resulted from fertilization has the totipotency to develop into a future seedling. Consequently, plants undergo global chromatin re-organization during sexual reproduction to develop into highly distinct cell types and establish cell pluri- or totipotency, in which the reprogramming of histone modifications plays a vital role (Kawashima and Berger, 2014; She and Baroux, 2014).

Plant sexual reproduction consists of three different phases: sporogenesis, gametogenesis, embryo- and endosperm-genesis. Plant reproduction initiates with sporogenesis, and it is characterized by the generation of meiotic-competent spore mother cells (SMCs), namely SMC differentiation (Kawashima and Berger, 2014; She and Baroux, 2014). The male SMCs, also known as pollen mother cells (PMCs) (*2n*), are differentiated in the sporangium and formed in the anther locule, then undergo meiosis to give rise to four haploid microspores (*1n*). After an asymmetric and atypical mitosis division, each microspore produces one vegetative cell with a larger nucleus (*1n*) and one generative cell with a smaller nucleus (*1n*). Subsequently, the generative cell (*1n*) undergoes one additional mitotic division to generate two sperm cells (*1n*). Mature pollen grain usually contains two sperm cells (*1n*) and one much larger vegetative cell (*1n*) (Twell, 2011). The female gametogenesis begins with the differentiation of the female SMCs, also called megaspore mother cells (MMCs), which occurs within the ovule primordia in the gynoecium. The MMCs (*2n*) undergo meiosis to generate four haploid spores (*1n*), while only one spore survives to form the functional megaspore cell (FMC) (*1n*). The FMC (*1n*) then undergoes three rounds of mitosis to develop into the eight-nucleated mature female gametophyte (embryo sac) consisting of one egg cell (*1n*), one central cell (*2n*), three antipodals (*1n*), two synergids (*1n*) (Drews and Koltunow, 2011; Baroux and Autran, 2015). All cells in the embryo sac are haploid except for the central cell that has a di-haploid maternal genome by inheriting two polar nuclei. Double fertilization is a unique fertilization feature for the angiosperms, and it is a process that two female gametes in the embryo sac, the egg cell (1*n*) and the central cell (2*n*), receive two sperm cells (1*n*) in a one-to-one manner to yield the diploid embryo and the triploid endosperm, respectively. These two fertilization products have distinct developmental fates (Bleckmann et al., 2014). The pre-embryo engages in a series of cell divisions to establish a mature embryo with the potential to develop into a future seedling, while the primary endosperm cell engages in a syncytial phase of proliferation to form an extra-embryonic nurturing tissue.

**FIGURE 3:**
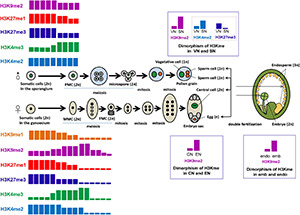
Dynamics of H3 lysine methylation during plant sexual reproduction. The process of plant sexual reproduction, including sporogenesis, gametogenesis, embryo- and endosperm-genesis, as well as the dynamics of H3 lysine methylation during plant sexual reproduction, are summarized in this figure. Differently colored rectangles represent different H3 methylation marks, including the orange rectangle for H3K9me1, the purple rectangle for H3K9me2, the red rectangle for H3K27me1, the blue rectangle for H3K27me3, the green rectangle for H3K4me3, and the light blue for H3K4me2. The height of the rectangles represents the relative enrichment of a certain methylation mark. In the flower, pollen mother cells (PMCs) (*2n*), differentiated from somatic cells in the sporangium, undergo meiosis to form four microspores (*1n*). Each microspore undergoes an asymmetrical division to generate one vegetative cell and one generative cell, which then divides to form two sperm cells (*1n*). The pair of sperm cells and the vegetative cell are linked to form the mature pollen grain. The sperm nucleus (SN) and vegetative nucleus (VN) have dimorphic pattern in their chromatin condensation, and the chromatin of SN is more compacted, accompanied by much more deposition of H3K9me2, compared with that in VN. Paradoxically, the transcriptionally permissive mark H3K4me2 is enriched and the repressive mark H3K27me3 is globally depleted in SN. The megaspore mother cells (MMCs) (*2n*) are generated from somatic cells in the gynoecium. MMCs undergo meiosis to generate four haploid spores (*1n*). The surviving megaspore, functional megaspore cell (FMC) (*1n*), undergoes three rounds of nuclear divisions to generate an eight-nuclei containing syncytial female gametophyte (embryo sac). After cytokinesis, the mature female gametophyte consists of the egg cell, the central cell, and accessory cells (antipodals and synergids). The egg nucleus (EN) exhibits more pronounced condensation, with significantly higher H3K9me2 deposition, compared to the central nucleus (CN). The egg cell and the central cell are each fertilized by one sperm cell to produce the diploid embryo (2n) and the triploid endosperm (3n), respectively. The dimorphic chromatin status present between the embryo (emb) and the endosperm (endo), which might be inherited from the female gametes after fertilization, with the embryo (emb) having highly condensed chromatin compared to the endosperm (endo), characterized by obviously lower H3K9me2 accumulation in emb than in endo.

Thereinafter, we will discuss the reprogramming of H3 lysine methylation and its molecular significance during sporogenesis, gametogenesis, embryo- and endosperm-genesis in flowering plants, mainly in *Arabidopsis*. The main process of sexual reproduction in plants and the dynamic regulation of H3 lysine methylation during these reproductive events are briefly summarized in Figure 3.

### Reprogramming of H3 Lysine Methylation During the Differentiation of Spore Mother Cells

The earliest event that occurs in sporogenesis is spore mother cells (SMCs) differentiation, which is usually characterized by histone modification-mediated chromatin reprogramming to contribute to the somatic-to-reproductive transition and the meiotic-competence cell fate establishment (Wang and Kohler, 2017; Gehring, 2019).

The differentiation of MMCs, the female SMCs, is marked by nuclear enlargement and chromatin decondensation, and this event coincides with a 60% reduction in heterochromatin content, a decreased number of chromocenters, and the depletion of canonical linker histone (She et al., 2013). Reprogramming of H3 lysine methylation contributes to the formation of the MMC-specific chromatin status, which helps to establish a transcriptionally permissive chromatin status (She et al., 2013). The MMC exhibits a 2.7-fold enrichment of the permissive-associated H3K4me3 mark and a 50% reduction of the repressive-related H3K27me3 mark (Berger and Twell, 2011; She et al., 2013), and some other repressive marks including H3K27me1 are also decreased in the MMC (Berger and Twell, 2011; She et al., 2013; She and Baroux, 2014). The reduction in H3K27me3 (relative to the increase of DNA content) at this pre-meiotic S-phase might be attributable to the non-methylated H3K27 residues in the newly generated nucleosomes by DNA replication. The increase of H3K4me3 mark during the S-phase and prophase I might activate some chromatin-modifying enzymes (She and Baroux, 2014). Strangely, the H3K4me2 level is reduced by 30% and the global H3K9me2 level has a 1.6-fold increase in the MMC (She et al., 2013), this seems inconsistent with the establishment of a permissive chromatin status in MMC as H3K4me2 is an active transcriptional mark while H3K9me2 is a repressive one.

As potentially mobile sequences within the genome, transposable elements (TEs) make it difficult for plants to transmit the genetic information accurately to the next generation. TEs are typically silenced by various repressive machineries including epigenetic modification. However, chromatin decondensation, heterochromatin reduction, and epigenetic reprogramming during plant sporogenesis provide a favorable probability for TEs to escape from silencing; therefore, it is of significance for plants to employ a series of strategies to restrict TEs movement, particularly in the germline (Bao and Yan, 2012). H3K9me2 is an important heterochromatic mark and plays a vital role in silencing the activities of TEs. During the transition of somatic cells to MMCs, H3K9me2 remains highly accumulated in chromocenters (She et al., 2013), suggesting a reinforcement of TEs silencing even though the heterochromatins are not maintained. Moreover, the increase in H3K9me2 levels might be highly specific as H3K27me1, another mark typically enriched at chromocenters, is reduced dramatically in the MMC heterochromatin. H3K9me2 seems to be accumulated by consuming H3K9me1 as H3K9me1 levels decrease in the MMC chromatin (She et al., 2013).

The development of male reproductive lineage begins with the differentiation of PMCs in the early anther locule. In *Arabidopsis*, one sub-epidermal somatic cell in the sporangium enlarges to form an archesporial cell, which then divides to generate a primary sporogenous cell, and subsequently, the sporogenous cell undergoes mitosis to give rise to PMCs. During PMC differentiation, nuclear morphology undergoes similar changes to that during MMC generation: the PMC exhibits a fivefold increase in nuclear volume size, accompanied by a decrease in heterochromatin content and the average number of distinct chromocenters. This indicates that a distinct nuclear organization related to the transcriptionally permissive chromatin landscape is developed in the PMC (She and Baroux, 2015). Specific histone modification reprogramming occurs to establish this distinct chromatin status in PMCs. The levels of two repressive marks H3K27me1 and H3K27me3 are decreased, while that of the permissive mark H3K4me3 has a 1.8-fold increase in PMCs compared to that in somatic cells. During the PMC differentiation, the level of H3K4me2 is constant compared to that in the surrounding somatic cells (She and Baroux, 2015), different from the decreased H3K4me2 level in MMCs. In PMCs, substantial normally silenced TEs become transcriptionally activated (Chen et al., 2010; Yang et al., 2012), indicating that the decondensation at heterochromatin loci can release some TEs silencing. H3K9me2 levels might be reduced before meiosis in PMCs as it acts to repress TE expression, which is different from the changes of H3K9me2 levels in MMCs. More detailed investigations remain necessary to reveal the dynamic events of histone lysine methylation underlying PMCs differentiation.

Collectively, both the female and male SMCs, MMCs, and PMCs, are competent in differentiating into several distinct cell types. Modulation of H3K27me1, H3K27me3, and H3K4me3 levels in mutants with altered gametophytic competence demonstrates the importance of H3 lysine methylation in pluripotency establishment during SMCs generation (Berr et al., 2010; Olmedo-Monfil et al., 2010). Moreover, the reprogramming of H3 lysine methylation might be a prerequisite for the subsequent meiosis as dynamic regulation of H3 lysine methylation at certain sites is critical for meiotic events, including homologous chromosome pairing, synapsis, and recombination initiation (She et al., 2013). Alternatively, the H3 lysine modification dynamic events might also contribute to activating the meiotic genes and repressing the mitotic pathway.

### Reprogramming of H3 Lysine Methylation During Gametogenesis

The female gametogenesis begins with meiosis initiation, then the formation of the eight-nuclei syncytium, and finally, the haploid egg cell and diploid central cell containing mature embryo sac are set definitively by cellularization. Meiotic execution requires additional dynamic histone modifications, particularly during prophase I with further enrichment of H3K4me3 marks occurring along the entire chromosomes (Baroux and Autran, 2015). Moreover, H3K27me1 level in the FMC chromatin is dramatically reduced, while H3K27me3 decreases to an undetectable level (She et al., 2013; Baroux and Autran, 2015). H3K9me2 level shows a more pronounced increase at prophase I during MMC differentiation, while decreases significantly in the FMC chromatin, implying that FMCs go through another wave of H3K9me2 organization (She et al., 2013). During three mitotic cycles of FMCs, the H3K9me2 mark is re-established to a higher level (Pillot et al., 2010).

Currently, histone modification reprogramming events underlying male gametogenesis are barely known, yet a dimorphic chromatin status is established between the sperm cells and the vegetative cell. The sperm cells have highly condensed chromatin, while the vegetative cell has highly decondensed chromatin (Schoft et al., 2009; She and Baroux, 2015).

### Dimorphic H3 Lysine Methylation States in Gametes and Their Companion Cells

As companion cells of gametes, the vegetative and central cells have largely decondensed chromatin, which functions importantly in maintaining the integrity of the germline genome and assisting the subsequent fertilization (Ibarra et al., 2012; Baroux and Autran, 2015). Moreover, the companion cells and their corresponding gametes are also marked with a stark dimorphism of the chromatin and transcriptional status, which involves not only unequal DNA methylation (reviewed in Han et al., 2019) but also a dimorphic H3 lysine methylation state. The companion cells have large decondensed chromatin, accompanied by an increase of transcriptionally active histone marks and a reduction of transcriptionally inactive histone marks (Pillot et al., 2010). Correspondingly, the repressive histone marks, including H3K9me2 deposition, are significantly reduced in the vegetative and central cells in both eudicots and monocot species (Baroux and Autran, 2015). The chromatin decondensation in companion cells seems to influence the epigenetic setup of the gamete cells. In the companion cells, massive transcription of TEs occurs (following active epigenetic marks) and then TE-specific siRNAs are generated as a consequence (Schoft et al., 2009; Slotkin et al., 2009). These siRNAs can travel into the corresponding gamete cell and act *in-trans* on the chromatin to reinforce TE silencing by inducing the RNA-directed DNA methylation pathway, indicative of the importance of companion cells in maintaining genome stability and integrity of the gametes (Ibarra et al., 2012). *Trans*-silencing of a reporter gene GFP was successfully achieved in the sperm cells by expressing a corresponding amiRNA in the vegetative cell. This result supports an idea that siRNA can move from the companion cell to the male gametes, and the mobility of siRNAs was also confirmed between the central cell and the egg cell (Ibarra et al., 2012; Feng et al., 2013).

In contrast to the companion cell chromatin, the gamete chromatin exhibits more pronounced condensation. The highly deposited H3K9me2 modification of the sperm chromatin, especially at heterochromatic loci, partially contributes to the condensed chromatin state. The egg cell chromatin also harbors much more H3K9me2 (Pillot et al., 2010; She and Baroux, 2014). Moreover, other repressive epigenetic marks and associated enzymes are also enriched in the egg cell; therefore, the transcription in the egg cell is almost at a quiescent state, coincident with low-to-undetectable levels of the active RNA Pol II (Pillot et al., 2010). Paradoxically, the transcriptionally permissive mark H3K4me2 is enriched and the repressive mark H3K27me3 is globally depleted in the sperm chromatin, which might be essential events for the transcription of sperm-specific genes (Pillot et al., 2010; She and Baroux, 2014). It is well-known that the transcriptomes of both the female and male gametes are characterized by a set of specifically expressed genes that are otherwise silenced in the somatic tissues. Thus, cell-specific epigenetic landscapes occurring during gametogenesis may create a favorable environment for the de-repression of those gamete-specific genes.

### Reprogramming of H3 Lysine Methylation During Fertilization and Pre-embryogenesis

In angiosperms, two fertilization products are generated following double fertilization, a specific process in which two haploid sperm cells (*1n*) are delivered to the embryo sac through the pollen tube and simultaneously to fertilize with the haploid egg cell (*1n*) and the homodiploid central cell (*2n*) to generate a diploid embryo (*2n*) and a triploid endosperm *(3n*), respectively (reviewed in Bleckmann et al., 2014). The dimorphic epigenetic chromatin status in the egg cell and central cell directly gives rise to the dimorphism of the chromatin and transcriptional status in these two fertilized products, the embryo (zygote) and the endosperm (Pillot et al., 2010). The highly permissive and transcriptionally active state of the central cell is largely inherited by the endosperm following fertilization, therefore the chromatin dynamics in the central cell is likely to be a pre-patterning event for its post-fertilization fate.

Genomic imprinting is the consequence of the dimorphic epigenetic status in the asymmetric epigenetic setup between the embryo and the endosperm (Rodrigues and Zilberman, 2015), and imprinting regulation involves PRC2-mediated histone modification and likely other epigenetic mechanisms. For instance, genes with permissive epigenetic marks in the central cell, but are highly repressed in the condensed sperm chromatin, will develop into maternally expressed imprinted genes (MEGs) in the endosperm after fertilization. The MEGs and the paternally-expressed imprinted genes (PEGs) associate closely with differentially methylated regions (DMRs) between the paternal and the maternal genome (Zhang et al., 2014; Wang et al., 2015; Wang and Kohler, 2017). The PRC2 complex can target these DMRs for H3K27me3, and it has been reported that H3K27me3 can be deposited at hypomethylated regions in the maternal genome to determine the imprinted expression of PEGs, indicating that paternally and maternally hypomethylated regions contribute to the silencing of neighboring genes (Hsieh et al., 2011; Wolff et al., 2011; Borg et al., 2020). Additionally, several MEGs in the endosperm, such as MEA and FIS2, are essential for seed development, and mutations in *MEA* and *FIS2* cause seed abortion after fertilization. A subset of PEGs has indeed been demonstrated to have functions in building interploidy hybridization barriers in *Arabidopsis* (Wolff et al., 2011; Kradolfer et al., 2013).

The pre-embryo seems in a quiescent transcriptional state with a barely detectable Pol II activity, while the endosperm harbors a transcriptionally active chromatin status as shown by abundant levels of engaged RNA Pol II (Pillot et al., 2010). The distinct chromatin and transcriptional states of the fertilized products are largely inherited from their female gametic progenitors, the egg cell or the central cell, thus H3K9me2 is enriched in the zygote but reduced in the endosperm. Similar to the situation in the gametes and their companion cells, the transcription of TEs in the endosperm is derepressed to produce TE specific siRNAs that travel into the embryo to reinforce TE silencing in the zygote (Mosher et al., 2009; Ibarra et al., 2012). The embryo has the potency to develop into a future plant to establish novel cell types and organ symmetries; therefore, the newly formed zygote must be released from the gametic programs to obtain totipotency. The rapid reprogramming of chromatin status and histone medication in the zygote might be required for the establishment of future totipotency.

## Discussion and Perspectives

Chromatin reprogramming during gametogenesis, fertilization, and early embryonic development is crucial not only in maintaining genomic integrity but also in setting pluri-or totipotency and resetting silenced genes necessary for the plant life cycle. Although some evidence has shown the existence of H3 lysine reprogramming during plant sexual reproduction, especially during cell fate specification, limitations in cell-specific epigenomic techniques still leave this exciting problem in a state of incomprehension. Great efforts are still required to overcome obstacles in cell-specific epigenomic profiling of the reproductive lineage, particularly in the model plant *Arabidopsis thaliana* with extremely small germ cells, zygote, and endosperm. The development of cell-specific nuclei isolation approaches, including INTACT (isolation of nuclei tagged in specific cell types) (Deal and Henikoff, 2011; Moreno-Romero et al., 2017) and FACS (fluorescence-activated cell sorting) (Moreno-Romero et al., 2017; Gustafsson et al., 2019; Nott et al., 2021), may prove to be a real asset in these efforts, though it still requires improvement in optimization. Single cell epigenomics is an inevitable solution to reveal the chromatin remodeling in multiple different cell types in the reproductive lineage, and to provide more accurate and integrated interpretation to fully understand the role of reprogramming events in functional gamete formation and seed development.

Epigenetic modification is a reversible mark, which can be removed from or redeposited to target genes to affect their expression. It will be of great interest to learn where, when, and how histone modification reprogramming occurs to reset the expression of those genes in different generations, two characteristic examples being the resetting of *FLOWERING LOCUS C* (*FLC*) (Sheldon et al., 2008) and miR156/157, the master regulator of vegetative phase change in plants (Nodine and Bartel, 2010). *FLC* expression is repressed mainly by VRN2-PRC2 mediated H3K27me3 deposition under vernalization or cold treatment until it is reset to an active transcriptional state during plant reproductive lineages. This off-reset process is mediated by depositing active epigenetic marks and by removing repressive marks (De Lucia et al., 2008; Sheldon et al., 2008; Liu et al., 2021). In the pro-embryo stage, a seed-specific pioneer transcription factor, the *LEAFY COTYLEDON1* (*LEC1*), has been shown to establish active chromatin modifications at the *FLC* locus to re-trigger *FLC* expression (Tao et al., 2017). In early embryogenesis, two homologous B3 domain transcription factors LEC2 and FUSCA3 (FUS3) compete against two repressive modifiers to disrupt *FLC* silencing (Tao et al., 2019). These results suggest that the mechanism of gene off-reset pattern can be revealed by identifying specific transcription factors or histone modification associated co-factors specifically expressed in distinct reproductive tissues, or by searching readers containing bivalent or multivalent histone mark recognizing domains. In addition, histone readers might also recruit or stabilize various transcription factors, chromatin remodeling complexes and other components of the transcriptional network at the chromatin level to ensure proper transcriptional outcomes. Therefore, identifying the reader proteins and related complex might provide insight into the mechanism of gene off-reset during plant reproduction. A typical off-on resetting pattern during the plant life cycle occurs in the regulation of miR156/157, which is highly expressed in the juvenile phase, but declines gradually in the adult phase. The temporal expression pattern of miR156/157 during vegetative development is shown to be a result of the removal of active epigenetic marks and the deposition of some repressive epigenetic marks (Wolff et al., 2015; Xu et al., 2016, 2018; Fouracre et al., 2021). It is reasonable to assume that the silenced miR156/miR157 should be re-activated in the gametogenesis or pre-embryo stage to maintain its higher expression level in the juvenile phase in the next generation.

The mechanism by which the cell receives the instruction during plant sexual reproduction to initiate histone modification reprogramming is still poorly understood, and how to finely regulate histone modification at specific time points in specific cells still remains an open scientific question. As various epigenetic events, especially histone modification and DNA methylation, cooperate and interplay with each other closely; therefore, revealing the reprogramming and initiation of DNA methylation and other epigenetic marks during sexual reproduction will provide meaningful references. In addition, a reasonable explanation is that specific histone modification associated enzymes are recruited by specific cofactors, binding effectors, or transcription factors, which might be exclusively expressed in special cell types or selectively expressed at a certain developmental point, to specific sites to initiate histone modification. Therefore, searching for the developmental stage-specific and cell type-specific transcription factors or associated co-factors for histone modifier recruitment to dynamically regulate the chromatin state at specific loci will be one of the major tasks in future research.

## Author Contributions

HF conceived the present idea and wrote the manuscript. HF and YS consulted and collected relevant references. GW supervised the project, provided critical feedback, and helped shape the final manuscript. GW and YS helped revise and proofread the manuscript. All authors discussed the results and commented on the manuscript.

## Conflict of Interest

The authors declare that the research was conducted in the absence of any commercial or financial relationships that could be construed as a potential conflict of interest.

## Publisher’s Note

All claims expressed in this article are solely those of the authors and do not necessarily represent those of their affiliated organizations, or those of the publisher, the editors and the reviewers. Any product that may be evaluated in this article, or claim that may be made by its manufacturer, is not guaranteed or endorsed by the publisher.

## Footnotes

1
www.chromDB.org
